# Novel Bandwidth Expander Supported Power Amplifier for Wideband Ultrasound Transducer Devices

**DOI:** 10.3390/s21072356

**Published:** 2021-03-28

**Authors:** Kyeongjin Kim, Hojong Choi

**Affiliations:** Department of Medical IT Convergence Engineering, Kumoh National Institute of Technology, 350-27 Gumi-daero, Gumi 39253, Korea; 20196092@kumoh.ac.kr

**Keywords:** bandwidth expander, ultrasound transducer device, power amplifier

## Abstract

Ultrasound transducer devices have their own frequency ranges, depending on the applications and specifications, due to penetration depth, sensitivity, and image resolution. For imaging applications, in particular, the transducer devices are preferable to have a wide bandwidth due to the specific information generated by the tissue or blood vessel structures. To support these ultrasound transducer devices, ultrasound power amplifier hardware with a wide bandwidth can improve the transducer performance. Therefore, we developed a new bandwidth expander circuit using specially designed switching architectures to increase the power amplifier bandwidth. The measured bandwidth of the power amplifier with the help of the bandwidth expander circuit increased by 56.9%. In addition, the measured echo bandwidths of the 15-, 20-, and 25-MHz transducer devices were increased by 8.1%, 6.0%, and 9.8%, respectively, with the help of the designed bandwidth expander circuit. Therefore, the designed architecture could help an ultrasound system hardware with a wider bandwidth, thus supporting the use of different frequency ultrasound transducer devices with a single developed ultrasound system.

## 1. Introduction

Ultrasound transducers are the main sensor devices used in ultrasound systems [1]. Different types of ultrasound transducers are used, depending on their applications and specifications [2]. Engineers have manufactured ultrasound transducers with the required diagnostic applications. For diagnostic applications with different positions and locations, the imaging resolution and sensitivity performance are merits in the evaluation of ultrasound systems [3]. Typically, lower-frequency ultrasound transducer devices have a higher penetration depth and lower imaging resolution than higher-frequency ultrasound transducer devices [4]. Smaller-sized ultrasound transducer devices for intracardiac and intravascular applications need to be used such that the penetration depth of the devices is lower, but a higher imaging resolution could be preferable [5].

**Figure 1:**
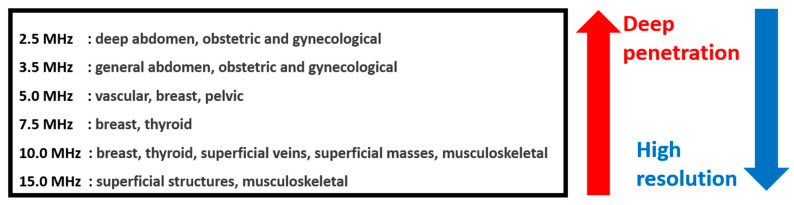
Penetration depth and imaging resolution for ultrasound diagnostic analysis.

Figure 1 shows the penetration depth and imaging resolution for an ultrasound diagnostic analysis. Since the imaging resolution and penetration depth have a trade-off relationship, ultrasound transducers with smaller piezoelectric elements are used depending on the body parts [6]. For the heart and abdomen areas, 2–3 and 3–5 MHz transducer devices are preferable because of the penetration depth [5,7]. For the eyeballs and breast and thyroid areas, 7.5–12 MHz and 7.5–13 MHz transducer devices are used due to their relatively high imaging resolutions [5,7]. In particular, for peripheral blood vessels and digestive tract areas, small transducer devices with a high frequency, such as 15–30 MHz, are used because of the higher imaging resolution, which reduces the penetration depth [8,9,10].

**Figure 2:**
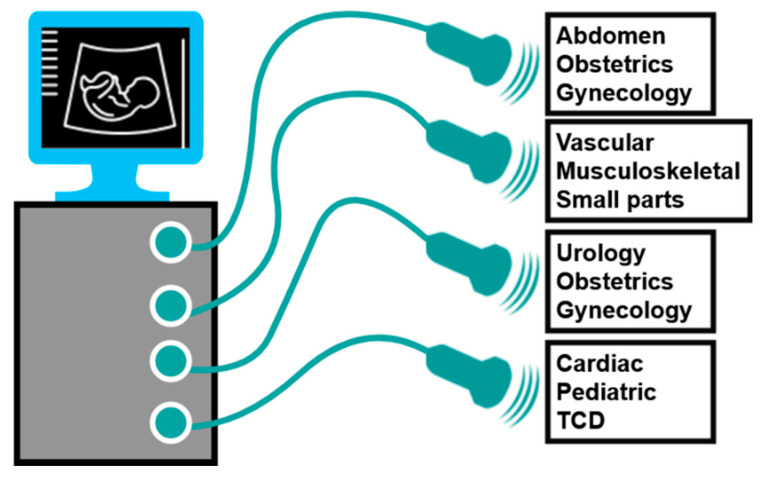
Transducer devices for various applications.

Figure 2 shows the ultrasound transducer probes (devices) used in ultrasound systems for specific applications [7,11]. Each ultrasound transducer probe has different frequency characteristics and shapes; thus, different anatomical cross-sectional images are obtained [11,12]. However, only one ultrasound system may not cover the transducer probes with different frequency ranges.

Various classes of power amplifiers have been developed for different types of ultrasound transducer devices. In power amplifiers, the input signals are amplified by active devices, which we call metal-oxide-semiconductor field-effect transistors (MOSFETs) or lateral-diffusion metal–oxide semiconductors (LDMOSs) [13]. Depending on the direct current (DC) bias voltages, the output currents generated by the active devices are fully or partially conducted during a single period of time [14]. This fundamental concept is used to categorize power amplifier classes, such as classes A, B, and C. In the Class A power amplifier, the operating bias voltage is located in the middle of the DC load line to minimize signal distortions [15]. Therefore, the active device in the power amplifier has a heavy load because the active device continues to operate regardless of the input signal [16,17]. Power is consumed continuously as long as an active device is operating. However, signal distortion by an active device is minimized, resulting in an output signal with high linearity [18]. In Class B power amplifiers, the bias voltage is located near the middle of the DC load line, and thus, the active device operates only for half the period of the signal [17]. Therefore, the active device operates for only half a period, which causes signal distortion, although the power consumption of the active device is reduced, compared to that of the Class A power amplifier. Class C power amplifiers operate for less than half a period of the signal because the bias voltage of the DC load line is lower than that of Class B amplifiers [13]. Therefore, the distortion of the signal is extremely high, whereas the power consumption of the active device is quite low. In addition, Class AB power amplifiers operate between Class A and B power amplifiers. In addition, there are other types of power amplifiers, such as Class D and Class E, which use harmonic components or modulated waveforms [19].

The Class B power amplifiers operate at only half of the pulse cycle, which has a much shorter time than Class A and Class AB power amplifiers. Therefore, Class B power amplifiers have much lower power consumption than those of Class A and Class AB power amplifiers, and thus, they have a higher efficiency [20]. The Class B power amplifier has lower signal distortions than the Class A power amplifier. Therefore, wire-type ultrasound machines have a Class A power amplifier because of the AC power cord [2]. Class B or Class C power amplifiers are preferable for mobile or portable ultrasound systems due to their limited battery modules [6]. Several studies related to power amplifiers have been conducted on ultrasound-transducer devices. For ultrasound signals, the burst or modulated waveforms with a limited time period and non-continuous signals are used. A typical Class A power amplifier was developed for ultrasound imaging applications [21]. A Class B power amplifier was developed to reduce the signal distortion [22].

There are a few studies of the power amplifiers used for ultrasound applications. There is a Class C power amplifier used for a 25 MHz transducer. This amplifier has high efficiency with high signal distortions. Therefore, the proposed circuit was developed to compensate for signal distortions [23]. Class D amplifier was developed for low-frequency piezoelectric transducers [24]. This amplifier was used for the high signal distortion and high-efficiency system. The Class E power amplifier was developed to improve the efficiency of low-frequency inductive piezoelectric converters [25]. As mentioned, various power amplifiers with various characteristics are used because all the amplifiers cannot satisfy some parameters of the signal distortion and the bandwidth. In ultrasound systems, transmitter and receiver architectures support ultrasound devices. The power amplifier in a transmitter is typically used in the last stage of electronics [2]. Thus, the bandwidth of the power amplifier only covers the specific transducer probes with their limited frequency ranges because the bandwidth of the power amplifier electronic devices decreases as the operating frequency of the power amplifier increases. This indicates that the output signals working at frequencies higher than the center frequency may deteriorate the image quality. Therefore, we first proposed a bandwidth-expander circuit for power amplifiers and various ultrasound devices.

There are several ways to increase the bandwidth of the power amplifier output. Impedance matching improves the amplitude and bandwidth of the power amplifiers and ultrasound transducers [26]. The output power can be increased through impedance matching, thus increasing the echo amplitude or the echo bandwidth of the ultrasound transducers [26]. However, the impedance of the ultrasound transducer varies significantly according to the frequency [27]. Furthermore, transducers are manufactured to have different central frequencies and resonance/anti-resonance frequency ranges [28]. Therefore, it can be difficult to match the impedance magnitudes within the desired frequency ranges for several ultrasound transducers. 

Another method is to lower the gain and increase the bandwidth of the power amplifier by using a feedback loop circuit [14]. This method a fundamental approach for increasing the bandwidth of a power amplifier. This method can reduce the gain of the power amplifier and increase the bandwidth [16]. Thus, several-stage power amplifiers need to be utilized to increase the gain and bandwidth. As the number of stages increases, various problems such as a time delay and signal distortions due to line resistances and parasitic nonlinear components could affect the performance of the power amplifiers [29]. Moreover, there could be a bandwidth expansion limit because space and cost are finite in terms of manufacturing.

In this study, we developed a circuit that can enlarge the bandwidth and minimize the signal loss, thus applying it to a single-stage power amplifier. In our proposed circuit, the bandwidth can be improved by lowering the input poles of the power amplifiers. The designed circuit works as a switching mode that is simply turned on/off with DC power. This method can be useful to improve the bandwidth of the power amplifier and minimize the signal loss, thus supporting higher-frequency ultrasound devices. Section 2 describes the theoretical background and analysis of the proposed circuit and the power amplifier. Section 3 presents the measured performance and a discussion of the proposed circuit with several ultrasound transducers. Section 4 provides some concluding remarks regarding this research.

## 2. Materials and Methods

The designed bandwidth expander (BWE) is a type of switching circuit operated by different applied DCs. It is located before the lateral-diffusion metal-oxide-semiconductor field-effect transistor (PD57018-E, LDMOSFET, STMicroelec-tronics, Geneva, Switzerland), which is a type of high-voltage MOSFET (BSS123, active device in BWE) in the input port of the amplifier. When the active LDMOSFET device in the amplifier operates under higher-voltage amplitudes than a certain bias voltage, the amplifier is applied to the gate of the LDMOSFET [30]. Therefore, the drain and source of the MOSFET are applied such that the input impedance of the amplifier is changed accordingly. In addition, by adjusting the input impedance, the input poles of the amplifier can be tuned. The total transfer function of the amplifier is expected to increase by integrating the BWE circuit with the amplifier. Therefore, the amplifier with the help of the BWE has a higher output amplitude and wider bandwidth.

### 2.1. Designed Power Amplifier and BWE Schematic Diagram

**Figure 3:**
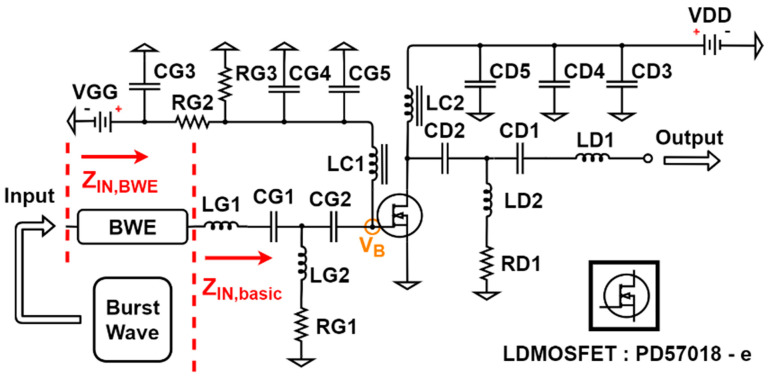
Designed amplifier schematic diagram.

Figure 3 shows an amplifier combined with a BWE circuit for the ultrasound transmitter. Table 1 shows the resistor, capacitor, and inductor elements of the amplifier, except for the LDMOSFET shown in Figure 3. The LDMOSFET (PD57018-E, STMicroelectronics, Geneva, Switzerland) was used as the core component of the amplifier. The simulation was conducted to make the impedance matching suitable for the 15 MHz transducer. In Figure 3, *V_B_* is the main gate-source operating point of the LDMOSFET. In addition, LC1 and LC2 are choke coil inductors that prevent the inflow of the alternating current (AC) into the DC power supply [31]. The components used in the input line (LG1, LG2, CG1, CG2, and *RG*1) and the components used in the output line (LD1, LD2, CD1, CD2, and RD1) are tuned to be compatible with the 15 MHz transducer and to achieve the values below −10 dB of S (1,1) and S (2,2). In addition, they need to block the DC current from *VGG* and VDD using CG2 and CD2. If AC input voltages enter the DC power supply, oscillations may occur [32]. The polarizing electrolytic capacitors CG3 and CD2 are helpful in providing a constant direct voltage [33]. The ceramic capacitors CG4, CG5, CD4, and CD5 were used to reduce noise as bypass capacitors. The bias voltage was adjusted to operate through the voltage distributions of *RG*2 and *RG*3. The bias voltage equation at *V_B_* is as follows [34,35]:

(1) $$V_{B}=VGG\times \frac{RG3}{RG2+RG3}.$$

In ultrasonic diagnostic equipment, the frequency of the input signal and the cycle of the burst wave were adjusted such that the Q factor used for providing an appropriate image quality was calculated [1]. Assuming that the LDMOSFET operates in the saturation region, the changes in the pole and transfer function can be estimated. As shown in Figure 3, the BWE circuit has little effect on the impedance. However, if a DC voltage is applied and operated, the input impedance changes. Therefore, the input pole changes. As the input pole is varied, the transfer function according to the frequency changes, and the output amplitude and bandwidth are changed. This concept is proved through several equations.

**Figure 4:**
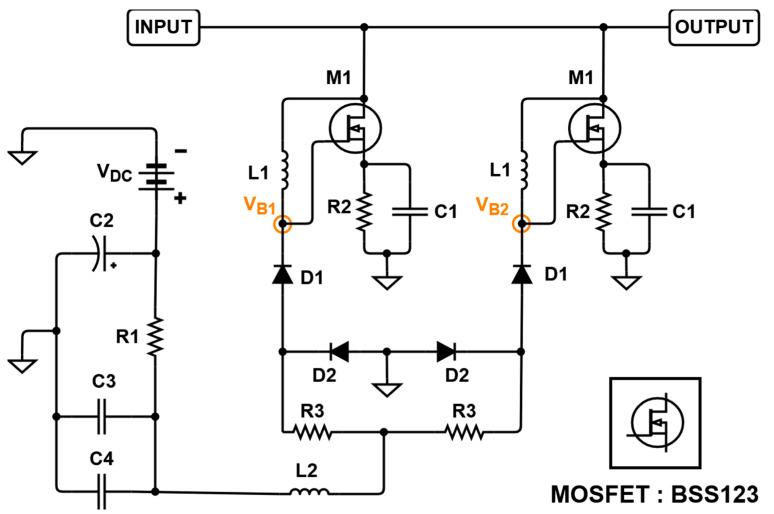
Schematic diagram of designed bandwidth expander (BWE) circuit.

Figure 4 shows a schematic diagram of the designed BWE to show the circuit element values. Table 2 shows the numerical values of the resistors, capacitors, and inductor elements in Figure 4. *R*1 and *R*3 were used to be tuned to have proper voltage distribution and power consumption because M1 needs to be operated properly. The input signal was connected to the gate of M1 to form a feedback loop and can cause oscillation to be blocked through L1. However, the too high value of inductance may distort the input signal, so we properly selected the inductor value. *R*2 and C1 play a major role to lower the input impedance because the drain and source of M1 are shorted. If the impedance is too low, signal amplitude can be reduced. Thus, we properly selected those values for an input signal of 15 MHz. This circuit is added to the circuit, as shown in Figure 3, and thus the amplifier performance by applying different constant DC voltages is changed depending on the MOSFET (M1, BSS123) operation. When this circuit is assumed to be an ideal current source or ideal switch, the DC bias voltage V_B1_ in the BWE circuit operates the transistor, M1, as described below [36,37,38]:

(2) $$V_{B1}=\left(V_{DC}-V_{D1\left(TH\right)}-V_{SD}\right)\times \frac{R_{2}||R_{2}}{R_{1}+R_{2}\left|\left|R_{2}+R_{3}\right|\right|R_{3}},$$

where *V_D_*_1(*TH*)_ and *V_SD_* are the threshold voltage and drain-source diode forward voltage, respectively. Above a certain voltage, *V_B_*_1_ shortens the drain and source of M1. As the drain and source voltages at M1 are short-circuited, C1, *R*2, and L1, and the capacitance and on-state resistance inside M1 are connected in parallel to the input impedance.

### 2.2. Predicting Performance Results 

**Figure 5:**
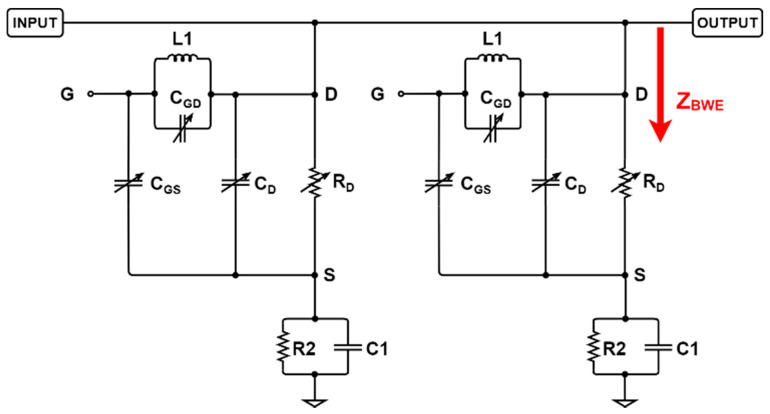
Equivalent circuit model when BWE circuit is operated.

Changes in the performance of the amplifier only and the amplifier with BWE can be expected and compared based on pole calculations. Therefore, the equivalent circuit models of the LDMOSFET in the amplifier and the MOSFET in the BWE were simplified to calculate the poles. First, the input impedance (*Z_IN_,_basic_*) of the amplifier (Figure 5) was calculated as follows:

(3) $$Z_{IN,basic}\;=\;X_{CG2}||\left(X_{LG2}+RG1\right)+X_{CG1}+X_{LG1},$$

where *X_CG_*_1_ and *X_CG_*_2_ are the impedances of the capacitors, and *X_LG_*_1_ is the impedance of the inductor (see Figure 5). When the *V_DC_* is applied to M1, the *V_PP_* of the input signal is blocked at D1 because the DC level is increased by V_B1_, as shown in Figure 4. In addition, the gate of M1 is short-circuited due to the applied V_DC_. The on-state resistance R_D_ and internal capacitances *C_GD_*, *C_GS_*, and *C_D_* exist. When M1 in the BWE circuit operates, it is expressed as an equivalent circuit for the AC analysis. Here, *Z_BWE_* indicates the impedance of the equivalent circuit when the circuit operates and is expressed as follows:

(4) $$Z_{BWE}\;=R_{D}\left|\left|X_{C_{D}}\right|\right|\left(X_{L1}||X_{C_{GD}}+X_{C_{GS}}\right)+R2||X_{C1}.$$

The input impedance in the BWE circuit was operated with parallel circuits, as shown in Figure 5.

(5) $$Z_{IN,\;\;BWE}=Z_{BWE}\left|\left|Z_{BWE}\right|\right|Z_{IN,basic}.$$

**Figure 6:**
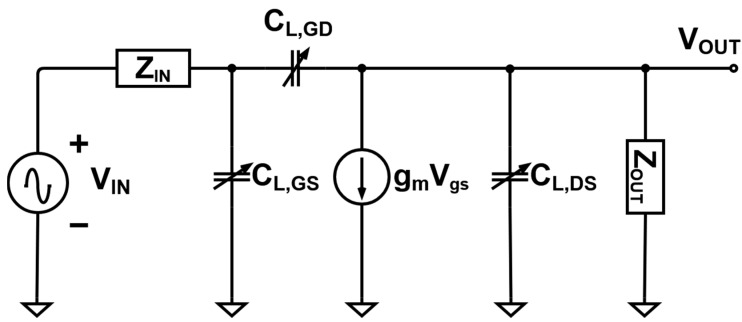
Equivalent circuit model of the amplifier.

Figure 6 shows the equivalent circuit model of the amplifier [17]. Assuming that the bias voltage *V_B_* of the amplifier only, and the amplifier with BWE are assumed to be the same, the internal capacitances *C_L,GD_*, *C_L,GS_*, *C_L,DS_*, and *g_m_V_gs_* are the same. 

As a result, the input and output poles and the transfer function can be predicted. The input pole of the amplifier only and the amplifier with the BWE circuit are given by Equation (6).

(6) $$\omega_{IN,basic}=\;\frac{1}{Z_{IN,basic}\left[C_{L,GS}+\left(1+g_{m}R_{D}\right)C_{GD}\right]}$$

(7) $$\omega_{IN,BWE}=\;\frac{1}{Z_{IN,BWE}\left[C_{L,GS}+\left(1+g_{m}R_{D}\right)C_{GD}\right]}$$

The output impedances of the amplifier only and the amplifier with the BWE circuit are assumed to be the same. The currents flowing from drain to source in the main transistor are the same if the same bias voltage is applied to both the amplifier and amplifier + *BWE*; the internal capacitance (*C_iss_*, *C_oss_*_,_ and *C_rss_*) is also the same. Thus, the internal capacitances (*C_L,GD_*, *C_L,GS_*, *C_L,DS_*, and *g_m_V_gs_*) are supposed to be the same. Therefore, the output impedances of the amplifier and amplifier + *BWE* are the same as *Z_OUT_*. Irrelevant to a transducer, the output signal does not change. Consequently, the output poles in the transfer functions of the amplifier only and the amplifier with the *BWE* circuit are expressed in Equations (8)–(10).

(8) $$\omega_{OUT}=\;\frac{1}{Z_{OUT}\left[C_{DS}+\left(1-A_{v}^{-1}\right)C_{GD}\right]}≅\frac{1}{Z_{OUT}\left(C_{DS}+C_{GD}\right)}$$

(9) $$\frac{V_{OUT}}{V_{IN}}\left(s\right),\;basic=\frac{-g_{m}Z_{OUT}}{\left(1+\frac{s}{\omega_{IN,basic}}\right)\left(1+\frac{s}{\omega_{OUT}}\right)}$$

(10) $$\frac{V_{OUT}}{V_{IN}}\left(s\right),\;BWE=\frac{-g_{m}Z_{OUT}}{\left(1+\frac{s}{\omega_{IN,\;BWE}}\right)\left(1+\frac{s}{\omega_{OUT}}\right)}$$

Equation (11) shows that *Z_IN,BWE_* has a relatively lower impedance than *Z_IN,basic_* because *Z_BWE_* is connected in parallel to *Z_IN,basic_*. Looking at Equations (6) and (7), the input pole has an inversely proportional relationship with the input impedance. Furthermore, from Equations (9) and (10), the transfer function has a proportional relationship with the input pole. As a result, the pole and transfer functions due to the different input impedances can be predicted as follows:

(11) $$Z_{IN,basic\;}>\;Z_{IN,BWE}$$

(12) $$\omega_{IN,basic\;}<\;\omega_{IN,BWE}$$

(13) $$\frac{V_{OUT}}{V_{IN}}\left(s\right),\;basic<\;\frac{V_{OUT}}{V_{IN}}\left(s\right),\;BWE.$$

However, the predicted results should be operated in an ideal environment. The actual results are extremely different from the predicted results because the high-voltage amplifier operations are not accurately predictable due to several high-voltage environment variables such as parasitic impedances, high-voltage valuable variances, and unpredictable equivalent inductance models [39,40].

**Figure 7:**
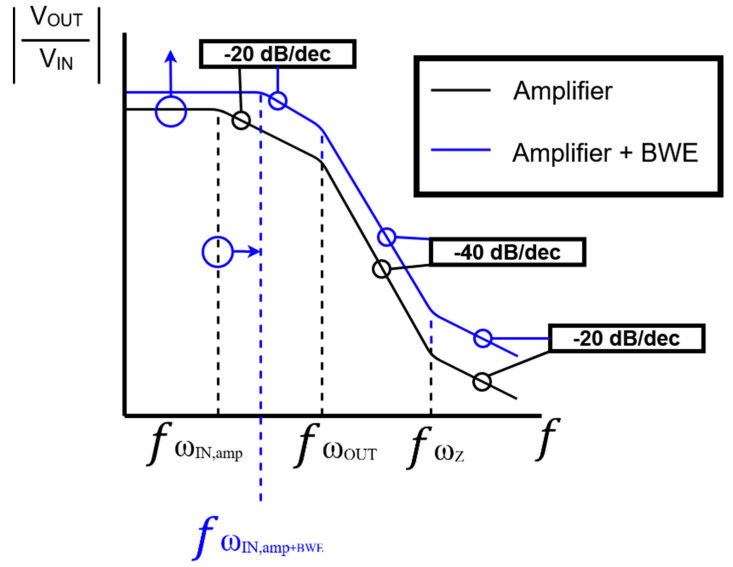
The predicted transfer function graph of the amplifier only and the amplifier with BWE.

Figure 7 shows the predicted graph based on Equations (11)–(13). By adding a *BWE* circuit to the existing amplifier, the magnitude and input pole in the transfer function increase. In Figure 7, the entire line goes up, and the input pole moves to the right. As a result, we assume that the line decreases by 20, 40, and 20 dB/dec, respectively, in the interval between the input pole, the output pole, and the zero point. Comparing the two cases, the transfer function is expected to increase. As the pole location shifts, we can expect to achieve a wider bandwidth.

### 2.3. Experimental Measurement Process

**Figure 8:**
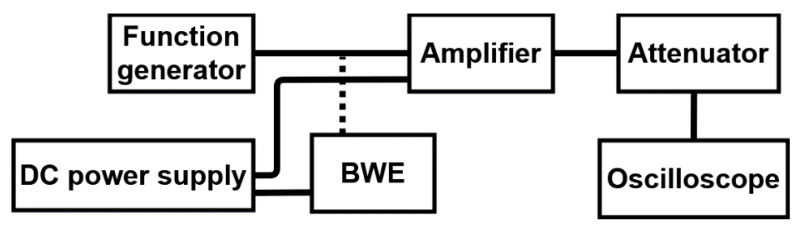
Block diagram showing the measured amplifier performances.

Figure 8 shows a block diagram showing the performance measurement of the amplifier only and the amplifier with *BWE* circuits. A function generator, DC power supply, attenuator, and oscilloscope were used to measure the performance of the designed circuits. A BWE circuit was installed between the function generator and amplifier. The BWE circuit operates when DC power is supplied by the power supply. The amplified signal can cause damage to the oscilloscope from a high voltage of 5 V_P-P_ or greater. Therefore, the output signals were attenuated by a 40 dB attenuator, and the performances were measured using an oscilloscope [41,42,43]. To measure the performance of the amplifiers, the frequency and input signal amplitudes were adjusted using a function generator. In addition, the voltage gain was obtained from the measured output signals. As a result, the outputs of the amplifier and BWE circuit-equipped amplifier were measured and compared. The voltage gain is a performance indicator for measuring the performance of an amplifier, and its high output helps to provide a clear ultrasound image [44,45,46]. In addition, the bandwidth of the gain over frequency of the amplifier only and the amplifier equipped with the BWE circuit can be compared. The output signal is an important performance indicator of an ultrasonic transmitter because it shows the sensitivity of the system.

**Figure 9:**
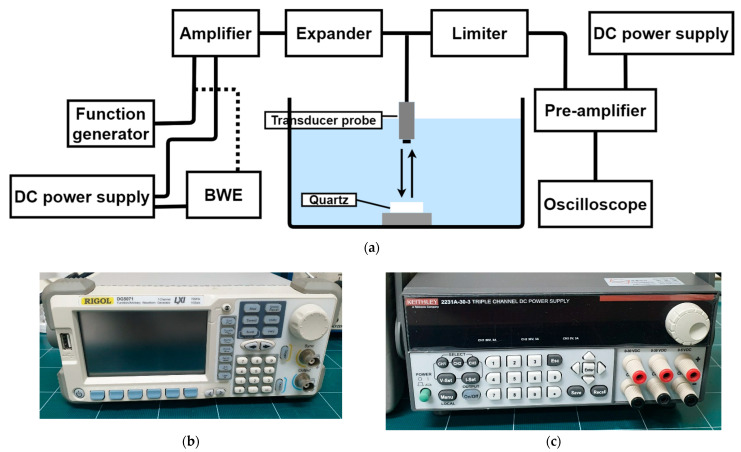

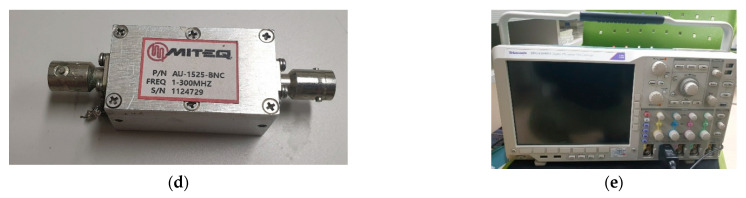
(**a**) Block diagram showing the measurement procedure of the echo signals using designed circuits and transducer probe, (**b**) function generator, (**c**) DC power supply, (**d**) pre-amplifier, and (**e**) oscilloscope.

Figure 9a shows the measurement procedures used to obtain the echo signal of the transducer with the designed amplifier with and without a BWE circuit. Various instruments have been used to measure the echo signal to determine its compatibility with ultrasonic transducer probes [47]. The amplified signal was passed through the expander [48,49]. The signal was transmitted through the transducer probe and reflected by the quartz to be received [50,51,52]. The expander was used to remove the noise and reduce the ringdown signal from amplified signals [53,54,55]. Since the received signal has an extremely low amplitude, it is amplified by an approximately 32 dB gain pre-amplifier and then displayed on the oscilloscope. During this process, because quartz reflects more than 99% of the signal, the data of the echo signal can be measured to estimate the amplifier performance [56,57]. The amplified signal, called a discharged signal, is required to vibrate the piezoelectric element of the transducer probe; however, it is not necessary to measure the echo signal, and the oscilloscope can be damaged with a voltage of higher than 5 V_P-P_, and thus, it is minimized using a limiter [58,59,60]. Figure 9a,b shows the tested equipment components used in Figure 9a.

**Figure 10:**
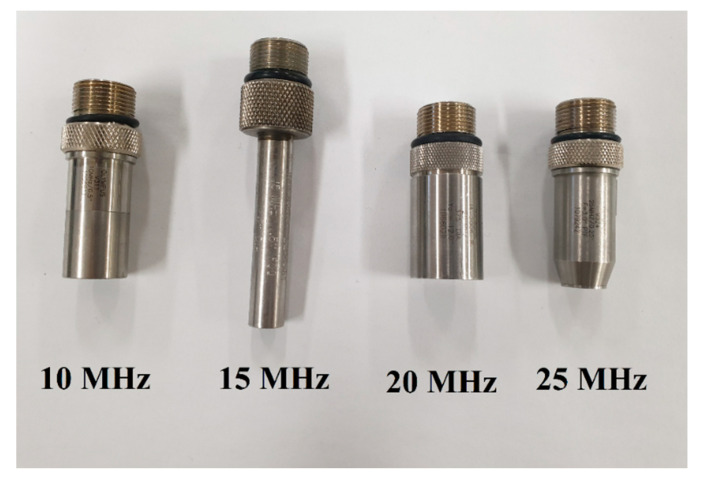
Transducer probes with each different frequency band used to measure the echo signals.

In Figure 10, the transducer probe was used to measure the echo signal to estimate the amplifier equipped with and without the BWE circuit. The amplifier performance was measured by adjusting the input signal frequency according to the resonance frequency of each transducer [61,62,63]. All input parameters are the same when the amplifier is equipped with and without the BWE circuit. The measured performances are the amplitudes and pulse widths of the echo signals, −6 dB bandwidths, and harmonic components using a fast Fourier transform (FFT). The harmonic distortion characteristics were estimated using the total harmonic distortion (THD) equation [64,65,66]:

(14) $$\mathrm{THD}=\;\frac{\sqrt{2ndharmonic^{2}+3rdharmonic^{2}}}{fundamental\;signal}$$

(15) THD (dB)=20logTHD,

where the second and third harmonics are the amplitudes of the second and third harmonic distortion components, and the fundamental signal is the amplitude of the fundamental signal at the desired operating frequency.

The higher the amplitude of the echo signal is, the higher the sensitivity of the transducer probe [47,67]. The narrower pulse width of the echo signal can result in a higher axial resolution of the transducer probe. The lateral resolution is related to the bandwidth [68]. The wider the bandwidth at the −6 dB point, the lower the Q factor, and thus, more image data can be realized [69]. However, the harmonic component generated unwanted image data [70,71]. Thus, these data need to be minimized. In this study, the measured performance factors were compared according to each transducer at different frequencies, as shown below. Therefore, the amplitudes, pulse widths, bandwidths, and THD were measured and compared by applying different transducer probes according to the frequency of each input signal with an amplifier equipped with and without the BWE circuit.

## 3. Results

**Figure 11:**
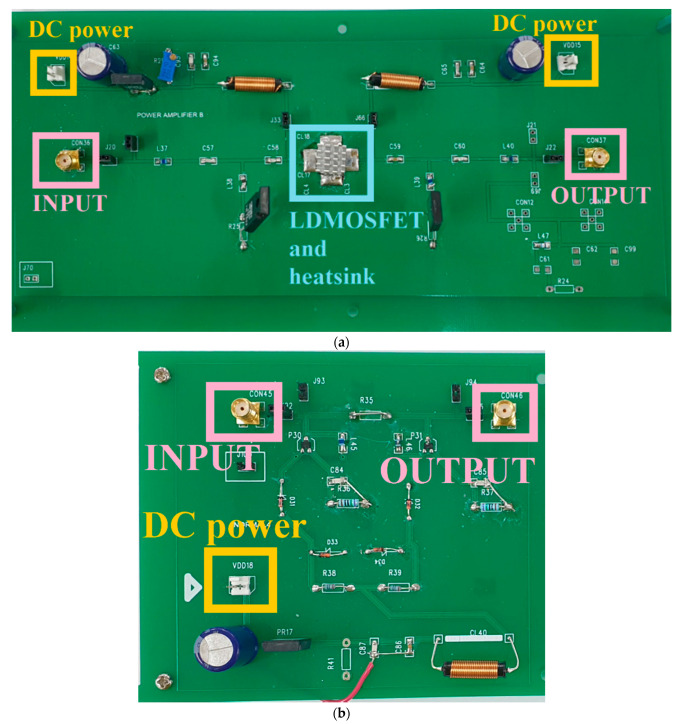
Manufactured (**a**) amplifier and (**b**) BWE circuits.

Figure 11a,b shows the manufactured single-ended power amplifier and BWE circuits, respectively. The main transistor (LDMOSFET) with a heatsink was used to release heat more effectively [72,73,74]. The output port in Figure 11b is connected to the input port, as shown in Figure 11a.

### 3.1. Performance Comparison and Analysis of the Amplifier Only and Amplifier + BWE Circuit

**Figure 12:**
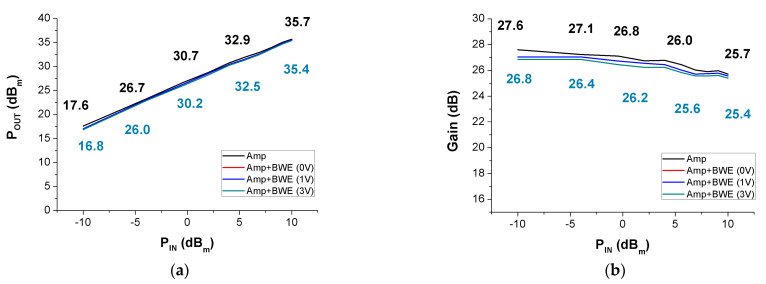

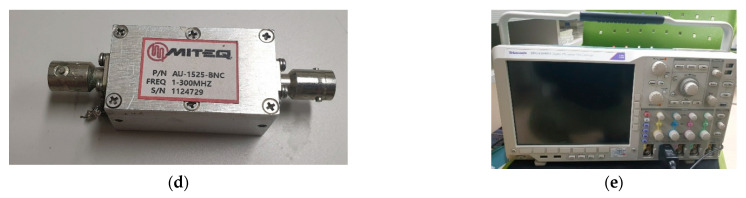
(**a**) P_out_ vs. P_IN_, (**b**) gain vs. P_IN_, (**c**) P_out_ vs. frequency, and (**d**) gain vs. frequency of the performance measurement results of the amplifier only and the amplifier with the addition of the BWE circuit.

Figure 12a,b shows the P_OUT_ and gain variances as the input signal increases. The black line shows the performance of the power amplifier. The red, blue, and khaki lines show the performance of the amplifier with the BWE circuit using 0V, 1V, and 3V DC, respectively. In the case of amp + *BWE* (0 V) and amp + *BWE* (1 V), the active device of the BWE (see M1 in Figure 4) is not in operation, and thus, they have almost the same performance. Therefore, the red line is almost identical to the blue line. In Figure 12a,b, the performance of the power amplifier has a higher P_OUT_ and gain than the amplifier with a BWE (3 V) of between −10 dB_m_ and 10 dB_m_.

Figure 12c,d shows the P_OUT_ and gain according to frequency variations at an input power of −6.5. In Figure 12c,d, the performances of the amplifier at 5–16 MHz have a higher P_OUT_ and gain than the power amplifier with BWE (3 V). The performance of the power amplifier with a BWE (3 V) has a higher P_OUT_ and gain of 17–35 MHz, compared to the power amplifier only. The P_OUT_ of the power amplifier with BWE (3V) outperformed that of the power amplifier after 16.132 MHz. In addition, the −3 dB P_OUT_ bandwidth of the power amplifier only and the power amplifier with BWE (3 V) were 51.5% and 81.5%, respectively. The −3 dB gain bandwidth of the power amplifier only and the power amplifier with BWE (3 V) are 84.1% and 141%, respectively. By incorporating the BWE circuit into the power amplifier, the P_OUT_ decreases to 0.8 dB_m_, and the gain decreases by 0.8 dB when the P_IN_ is −10 dB_m_. Theoretically, the magnitude of the transfer function increases with the addition of the BWE circuit to the power amplifier. Although the P_OUT_ and gain should be increased together, in practice, the final output signal can be slightly reduced because of the power loss of the passive components in the BWE circuit. However, in the graphs of the P_OUT_ and gain versus frequency, the P_OUT_ bandwidth increases by approximately 30%, and the gain bandwidth increases by approximately 56.9%. In this paper, the BWE circuit is used to widen the bandwidth by lowering the input impedance of the power amplifier. However, it does not decrease input impedance linearly at all frequencies. As shown in the experimental results, the bandwidth at the high-frequency range is wider than that of the low-frequency range because the BWE circuit has more impedance reduction at high frequency (See Figure 12c,d)

**Figure 13:**
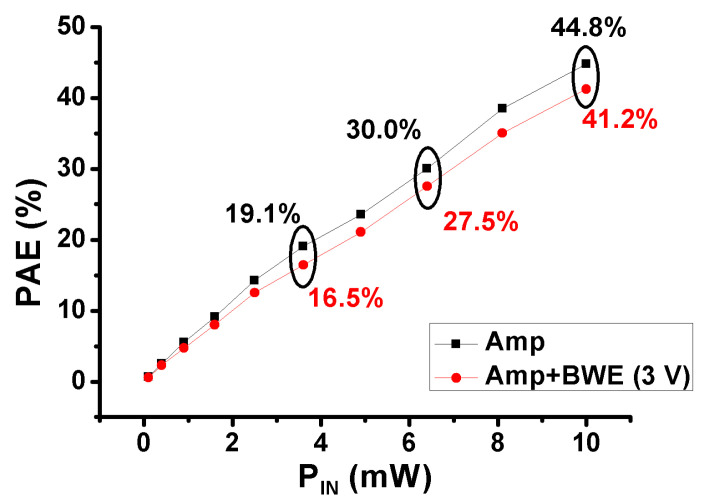
Power added efficiency (PAE) vs. P_IN_ measured results of the amplifier only and the amplifier with the addition of the BWE circuit.

Figure 13 is the graph of the power added efficiency (PAE) of the amp and the amp + BWE (3 V) at 15 MHz. The PAE indicates how much DC power was used to amplify the input signal [15,75]. In Figure 13, the PAE versus P_IN_ of the amp (44.8%) is higher than the amp + BWE (3 V) (41.2%) when 10 dB_m_ input power is applied. This is because the additional DC power is used for the designed BWE, and the amp has a higher P_OUT_ versus the P_IN_ than the amp + BWE (3 V). As a result, the PAEs of the amp and the amp + BWE (3V) do not show a big difference between them.

### 3.2. Echo Signal Performance Comparison and Analysis

**Figure 14:**
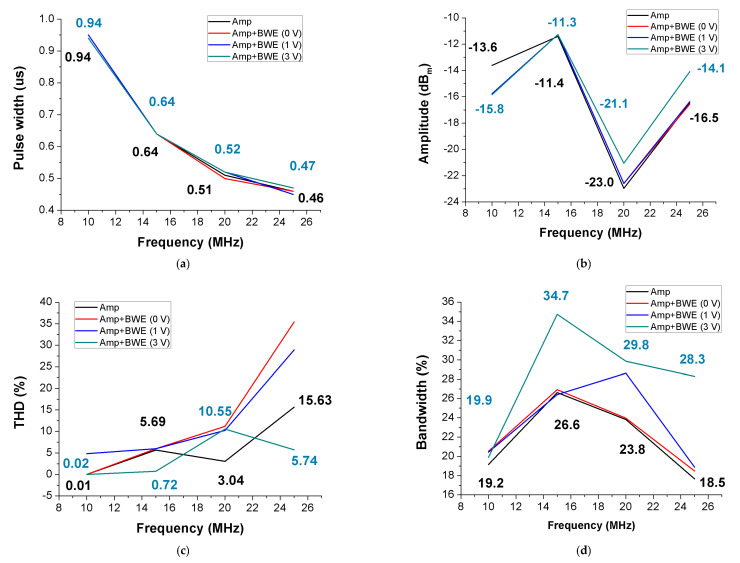
(**a**) Pulse width vs. frequency, (**b**) amplitude vs. frequency, (**c**) total harmonic distortion (THD) vs. frequency, and (**d**) bandwidth vs. frequency of the measured echo signal data using 10, 15, 20, and 25 MHz ultrasonic probes.

Figure 14 shows measured echo signal performances when using 10, 15, 20, and 25 MHz ultrasound transducers. When the ultrasound transducers with the same frequency were used, the distance to the target was exactly the same. The input signal was measured using a four-cycle burst wave with a suitable resonant frequency for the transducer probes. The measurement environment of each frequency is the same except for the presence of BWE with different DC voltages. As shown in Figure 14, the red, blue, and khaki lines show the performance of the power amplifier only and the power amplifier with BWE circuit using 0 V, 1 V, and 3V DC, respectively. In the case of the amplifier + *BWE* (0 V) and amplifier + *BWE* (1 V), the MOSFET (referring to M1 in Figure 4), which is the active device of the BWE circuit, is not operated and thus shows almost the same performances.

Figure 14a,b shows the measured results of the pulse widths and amplitudes over the time scale. Figure 14a shows the pulse width according to the frequency. The experimental results showed no significant difference in any of the measured frequency bands. Experimentally, the BWE circuit does not have a significant influence on the pulse width of the echo signals. Figure 14b shows the measured echo amplitudes of the peak-to-peak voltage according to the frequency using a 32 dB preamplifier. As shown in the graph, the amplitude of amp + *BWE* (3 V) was higher than that of the amp after 15 MHz. At 25 MHz, there is a difference of approximately 2.4 dB_m_. Since the echo signal has an extremely low amplitude, a 2.4 dB_m_ increment in the amplitude is an attractive result.

Figure 14c,d shows the calculated FFT data used to measure the harmonics and −6 dB bandwidths of the measured echo signals. Figure 14c shows the THD (%) according to the frequency. At 20 MHz, the THD of the amp was calculated as 7.15%, which is less than that of the amp + *BWE* (3 V). However, at 25 MHz, the THD of the amp was calculated as 15.63%, and that of the amp + *BWE* (3 V) was calculated as 5.74%. Figure 14d shows the echo bandwidth according to the frequency. By adding a BWE (3 V) circuit to the power amplifier, the −6 dB bandwidth of the echo signal was increased by 0.7%, 8.1%, 6.0%, and 9.8% at 10, 15, 20, and 25 MHz, respectively. The bandwidth of the echo signal is actually related to the axial resolution of the ultrasound image, and thus, a wider bandwidth can possibly improve the axial resolution [11]. By adding a *BWE* circuit to the power amplifier, the bandwidth of the echo signal is increased, which can help improve the quality of the echo signals.

Both low and high-frequency transducers can be utilized by using the proposed BWE circuit with the amplifier. There are some ultrasound applications that utilize dual-band transducer applications [76]. For these applications, the signals of low- and high-frequency ranges from dual-band therapeutic/imaging transducer applications need to be obtained. From the paper, the ultrasound transducers enable therapeutic and imaging modes if needed. A treatment application requires to use many cycle sinusoidal waveforms [77]. The harmonic components generated when amplifying the input signal can affect the signal quality of the echo signals [78]. For low-frequency therapeutic applications, the harmonic components can distort the signal and attenuate the depth of penetration [79]. Therefore, the amplifiers used for therapeutic applications are preferred to have a narrow bandwidth in order to minimize harmonic components. For high-frequency imaging applications, the wider bandwidth, the higher axial resolution can be achieved [80]. Therefore, an amplifier with wide bandwidth is preferred. Hence, our proposed scheme could be useful for such dual-band transducer applications.

## 4. Conclusions

The transducers used in ultrasound systems have their own different frequency bands, depending on the particular purpose and testing area. Therefore, it is necessary to use an electrical circuit with a wide bandwidth such that the output signal of the transmitter can cover various ultrasound transducers. One way of expanding the bandwidth is impedance matching. Impedance matching is required to maximize the amplitudes or bandwidths of the output signals to the transducer. However, an impedance-matching job that can cover such wide transducers is extremely difficult because the impedance is different for each transducer. Although impedance matching is not taken into account in this document, it is clear that impedance matching can be used if we know the impedance values of the predetermined transducer. However, this method used to increase the bandwidth could possibly lower the output amplitude at the center frequency. In addition, the feedback loop circuit methodology can increase the bandwidth by reducing the output amplitude of the power amplifier. Therefore, we propose a switching mode transmit circuit that can widen the bandwidth and minimize the output amplitude as needed. The designed BWE circuit changes the performance of the power amplifier because the bandwidth in the transfer function is widened by moving the input pole of the power amplifier.

To verify our proposed concept and verify the performance results, we tested a power amplifier equipped with a BWE circuit under the same conditions. Comparing the performances of the manufactured amplifier only and the amplifier with the BWE circuit, the P_OUT_ and gain values of the amplifier with the BWE circuit were decreased slightly to 0.8 dB_m_ and 0.8 dB; however, the P_OUT_ bandwidth increased by approximately 30%, and the gain bandwidth increased by approximately 56.9% at −6.5 dB_m_ of input power. In addition, the echo bandwidths were expanded by 0.7%, 8.1%, 6.0%, and 9.8% at frequencies of 10, 15, 20, and 25 MHz, respectively.

In practice, the measured experimental data may be different from the theoretical data because there are various side effects caused by signal distortions of different frequency characteristics and parasitic components of the elements. From the experimental results, none of the measured performances were enhanced when adding a functional BWE circuit. Although the bandwidth is wider, there is a slight compromise, such as a decline in output power or an increase in THD (%). However, the manufactured BWE circuit improves the bandwidth and minimizes the amplitude of the power amplifier to support higher operating transducer probes, thus possibly helping improve the ultrasound system resolution.

## Figures and Tables

**Table 1:** Numerical values of the circuit elements of Figure 3.

Components	Values	Components	Values
*RG*1	200 ohm	CD2	850 µF
*RG*2	1000 ohm	CD3	220 µF
*RG*3	Variable resistance	CD4	1000 pF
RD1	200 ohm	CD5	100 pF
CG1	550 pF	LG1	21 nH
CG2	340 pF	LG2	1000 nH
CG3	220 µF	LD1	130 nH
CG4	1000 pF	LD2	500 nH
CG5	100 pF	LC1	1 µH
CD1	340 pF	LC2	1 µH

**Table 2:** Numerical values of the circuit elements of Figure 4.

Components	Values	Components	Values
*R*1	150 ohm	C3	1000 pF
*R*2	750 ohm	C4	100 pF
*R*3	50 ohm	L1	560 nH
C1	47 pF	L2	1 µH
C2	220 µF		

## Data Availability

The data presented in this study are available on request from the corresponding author.
